# Dr. Harrison, on Vaccine Inoculation

**Published:** 1801-02

**Authors:** J. Banks, E. Harrison

**Affiliations:** Soho Square; Horncastle


### To Dr. BATTY.

DEAR SIR,

THE enclofed letter from Dr. Harrifon appears to me interefting to the public, and particularly fo to thofe who are friends to the Vaccine Inoculation; if you think it proper for your publication it will gratify me to fee it there, and I am fure it will alfo be agreeable to our friend Dr. Harrifon. I am,

DEAR SIR,

With real efteem and regard, your very humble fervant,

*Soho Square, Jan.* 13, 1801. J. BANKS.

---

### To the Right. Hon. Sir J. BANKS, K. B.

SIR,

I received a letter laft night from a correfpondent in London, who informs me that you had expreffed a defire to be made acquainted with the particulars of a cafe of Cow-pox Inoculation, which occurred in this neighbourhood, and fix months afterwards was followed by the Small-pox.

Upwards

Upwards of twelve months ſince, the nurſe-maid, and two children of my friend, the Rev. Marmaduke Allington, of Swinop-Houſe, were inoculated for the Cow-pox, with matter which had been ſent from a great diſtance. Neither of the infants were infected, but the maid had conſiderable inflammation on the arm, and although it was not attended with indiſpoſition, ſhe remains inſenſible to the variolous impreſſion. From the nurſe were inoculated a female ſervant, a third child, and Miſs Fanny Allington, who was then about five months old, had the operation repeated. The ſervant did not take the Cow-pox, but the children were ſuppoſed at the time to paſs through it in an eaſy manner. Though I did not ſee any of the patients before they were inoculated for the Small-pox, I had frequent converſations upon the ſubject with the attending ſurgeon, Mr. Sexty, who was always doubtful and diſſatiſfied with the experiments. From him, and ſome other reſpectable friends, who have carefully enquired into the phenomena, I am informed, that in Fanny's arm the progreſs of the puſtule was unuſually rapid, and was characterized by an areola that was neither extenſive nor circumſcribed. Mr. Sexty is an accurate and reſpectable practitioner, but, on account of other avocations and his diſtant reſidence, the patients were only viſited occaſionally; and as there was no conſtitutional indiſpoſition, Mr. Allington paid them no particular attention. From theſe cauſes the appearances on the inciſed parts cannot be minutely detailed, but I think a ſufficient number of circumſtances may be collected, to enable us to come to a ſatiſfactory concluſion on the ſubject. In both children the inciſed parts were inflamed, and a fluid was produced, but they had neither eruption nor illneſs. With matter taken from the arm of Fanny, the maid was again expoſed to the vaccine diſorder. From her ſeveral others were infected, and when the Cow-pox had been propagated through different ſubjects, Mr. Allington's other child was likewiſe inoculated. Six months afterwards they were all expoſed to variolous inoculation, and Fanny took the diſorder. She had a mild Small-pox with a moderate eruption. Hence it appears, that Fanny communicated a ſecurity againſt the Small-pox to others, although ſhe herſelf remained liable to its influence.

Several well atteſted inſtances are upon record, where variolous contagion from inoculated puſtules has produced a perfect Small-pox, and yet the ſubjects from whoſe arms it was taken had only a local infection, and afterwards paſſed through the Small-pox, either in the natural way or by inoculation. If it were neceſſary, I could corroborate this fact by detailing ſeveral caſes, which either occurred in my own practice, or have been been communicated to me by gentlemen of veracity and judgment.

In the prefent inftance I cannot undertake to determine, whether the failure was owing to a *peculiarity in the child's conftitution, or to the matter with which fhe was inoculated.* When the variolous, or vaccine, contagions are carelefsly dried and preferved, or are taken from old puftules, the nature of the poifon is fometimes fo much changed, that it neither produces its peculiar difeafe, nor does it afford any real fecurity. In other cafes, where a variolous puftule arifes upon the inoculated part, and paffes through its different ftages without producing any general infection, the failure is not to be imputed to the matter employed. In fuch inftances it muft have been occafioned by fome unfufceptibility, or unfitnefs to receive the peculiar impreffion. Nurfes are liable to variolous eruptions, from attending upon children in that complaint. With them they are purely local; and when perfons who have never had the Small-pox or vaccine diforders, remain proof againft infection, it arifes from fome conftitutional peculiarity, which takes place at the time, and with which we are juft as much acquainted as with the change that is produced by the action of variolous or vaccine matter upon the general habit. Sometimes, though rarely, this difpofition continues through life; at other times it affords only a temporary protection; and it is worthy of remark, that cafes have occurred where the fame perfon has been twice affected with the Small-pox.

Experienced practitioners are enabled to determine with confidence, whether the patient will continue to be exempt from the Vaccine and Small-pox Difeafes, by obferving the appearances that take place in the inoculated arm. When a fluid is produced too foon after inoculation; when the pimple is too much elevated, has no areola, or only a fmall and irregularly inflamed margin; or when it is accompanied by confiderable efflorefcence, and forms a fcab before the ufual time, there is reafon to conclude the perfon has only gone through the local complaint, and has received no protection againft the confequences of any future expofures.

Whether the failure in this cafe is to be imputed to the matter employed, or to the child's habit of body, it affords no folid objection to Vaccine Inoculation, fince the fame want of fuccefs has occurred in the Small-pox. It tends, however, to confirm the clofe analogy that has been found to fubfift between thefe two difeafes; and although, in the infancy of enquiry, it may be better to record facts than indulge in fpeculations, I am inclined to believe, the more we become acquainted with their their laws, the greater will be found the refemblance between them.

I have the honour to be, with great refpect and efteem,

Sir,

Your much obliged,

And faithful humble fervant,

*Horncaftle, Dec.* 30, 1800. E. HARRISON.

*P. S.* About two years fince we inoculated nearly one hundred perfons for the Cow-pox, with matter which you had obligingly provided for the Public Difpenfary. In one inftance, a fmall tuft of hair, refembling that on the child's head, was obferved to grow from the incifed part immediately after the defquammation.

